# Primary Human Osteoblasts in Response to 25-Hydroxyvitamin D_3_, 1,25-Dihydroxyvitamin D_3_ and 24R,25-Dihydroxyvitamin D_3_

**DOI:** 10.1371/journal.pone.0110283

**Published:** 2014-10-17

**Authors:** Karen van der Meijden, Paul Lips, Marjolein van Driel, Annemieke C. Heijboer, Engelbert A. J. M. Schulten, Martin den Heijer, Nathalie Bravenboer

**Affiliations:** 1 Department of Internal Medicine/Endocrinology, VU University Medical Center, Research Institute MOVE, Amsterdam, The Netherlands; 2 Department of Internal Medicine/Endocrinology, Erasmus Medical Center, Rotterdam, The Netherlands; 3 Department of Clinical Chemistry, VU University Medical Center, Research Institute MOVE, Amsterdam, The Netherlands; 4 Department of Oral and Maxillofacial Surgery/Oral Pathology, VU University Medical Center, Academic Centre for Dentistry Amsterdam, Amsterdam, The Netherlands; University of Tennessee, United States of America

## Abstract

The most biologically active metabolite 1,25-dihydroxyvitamin D_3_ (1,25(OH)_2_D_3_) has well known direct effects on osteoblast growth and differentiation *in vitro*. The precursor 25-hydroxyvitamin D_3_ (25(OH)D_3_) can affect osteoblast function via conversion to 1,25(OH)_2_D_3_, however, it is largely unknown whether 25(OH)D_3_ can affect primary osteoblast function on its own. Furthermore, 25(OH)D_3_ is not only converted to 1,25(OH)_2_D_3_, but also to 24R,25-dihydroxyvitamin D_3_ (24R,25(OH)_2_D_3_) which may have bioactivity as well. Therefore we used a primary human osteoblast model to examine whether 25(OH)D_3_ itself can affect osteoblast function using CYP27B1 silencing and to investigate whether 24R,25(OH)_2_D_3_ can affect osteoblast function. We showed that primary human osteoblasts responded to both 25(OH)D_3_ and 1,25(OH)_2_D_3_ by reducing their proliferation and enhancing their differentiation by the increase of alkaline phosphatase, osteocalcin and osteopontin expression. Osteoblasts expressed CYP27B1 and CYP24 and synthesized 1,25(OH)_2_D_3_ and 24R,25(OH)_2_D_3_ dose-dependently. Silencing of CYP27B1 resulted in a decline of 1,25(OH)_2_D_3_ synthesis, but we observed no significant differences in mRNA levels of differentiation markers in CYP27B1-silenced cells compared to control cells after treatment with 25(OH)D_3_. We demonstrated that 24R,25(OH)_2_D_3_ increased mRNA levels of alkaline phosphatase, osteocalcin and osteopontin. In addition, 24R,25(OH)_2_D_3_ strongly increased CYP24 mRNA. In conclusion, the vitamin D metabolites 25(OH)D_3_, 1,25(OH)_2_D_3_ and 24R,25(OH)_2_D_3_ can affect osteoblast differentiation directly or indirectly. We showed that primary human osteoblasts not only respond to 1,25(OH)_2_D_3_, but also to 24R,25(OH)_2_D_3_ by enhancing osteoblast differentiation. This suggests that 25(OH)D_3_ can affect osteoblast differentiation via conversion to the active metabolite 1,25(OH)_2_D_3_, but also via conversion to 24R,25(OH)_2_D_3_. Whether 25(OH)D_3_ has direct actions on osteoblast function needs further investigation.

## Introduction

Vitamin D deficiency, a common condition in the elderly population, has been associated to numerous skeletal health problems. Vitamin D deficiency causes a decrease of calcium absorption from the intestines and secondary hyperparathyroidism which leads to bone loss, osteoporosis and mineralization defects in the long term [1]. Vitamin D status is determined by the measurement of the metabolite 25-hydroxyvitamin D_3_ (25(OH)D_3_) [2], which is the major circulating form of vitamin D. The metabolite 25(OH)D_3_ is metabolized in the kidney by the enzyme 1α-hydroxylase (CYP27B1) into the biologically most active metabolite 1,25-dihydroxyvitamin D_3_ (1,25(OH)_2_D_3_) [3], which is the classical pathway for vitamin D activation. Both 25(OH)D_3_ and 1,25(OH)_2_D_3_ are metabolized by the enzyme 24-hydroxylase (CYP24), responsible for the first step in the inactivation process, to respectively 24R,25-dihydroxyvitamin D_3_ (24R,25(OH)_2_D_3_) and 1,24R,25-trihydroxyvitamin D_3_ (1,24R,25(OH)_3_D_3_) [4]. In addition, alternative pathways for vitamin D activation have been described, and one of such is the CYP11A1-mediated pathway [5]. This pathway for activation of vitamin D has been demonstrated in placentas *ex utero*, adrenal glands *ex vivo* and in cultured epidermal keratinocytes and colonic Caco-2 cells [6], [7]. Hydroxyvitamin D derivatives synthesized by the action of CYP11A1 not only act on the vitamin D receptor (VDR), but also on the retinoic acid related receptors α and γ (RORα and RORγ) [8].

The metabolite 1,25(OH)_2_D_3_ exerts its function by binding to the VDR which is present in numerous tissues, including bone tissue [3]. Bone formation is affected by 1,25(OH)_2_D_3_ both in an indirect and direct manner. Indirect effects of 1,25(OH)_2_D_3_ occur through stimulation of intestinal calcium absorption required for the maintenance of normal serum calcium levels and bone mineralization [3]. Direct effects of 1,25(OH)_2_D_3_ on osteoblasts have been demonstrated *in vitro.* These *in vitro* studies show that 1,25(OH)_2_D_3_ decreases osteoblast proliferation and stimulates osteoblast differentiation by increasing collagen type I synthesis and by secreting several non-collagenous proteins, for example osteocalcin and osteopontin [9]. The metabolite 1,25(OH)_2_D_3_ also increases the alkaline phosphatase (ALP) activity and the mineralization of bone matrix synthesized by human osteoblasts [10]–[12].

While the effects of 1,25(OH)_2_D_3_ on human osteoblasts are well-known, fewer studies have focused on the response of human osteoblasts to the precursor 25(OH)D_3_. Van Driel et al [12] have shown that 25(OH)D_3_ increases the ALP activity, the osteocalcin expression, and the early phase of mineralization in the human SV-HFO cell line. In primary osteoblasts, 25(OH)D_3_ inhibits the proliferation, stimulates the expression of osteocalcin and osteopontin, and increases the mineralization [13]. The actions of 25(OH)D_3_ on human osteoblasts are thought to take place after its conversion to 1,25(OH)_2_D_3_, since osteoblasts express 1α-hydroxylase and are capable of synthesizing 1,25(OH)_2_D_3_ from 25(OH)D_3_ [12]–[14]. Locally synthesized 1,25(OH)_2_D_3_ is thought to act in an autocrine or paracrine manner to regulate osteoblast proliferation and differentiation [12], [13]. However, it is largely unknown whether, in addition to the effects of 25(OH)D_3_ that occur via hydroxylation to 1,25(OH)_2_D_3_, 25(OH)D_3_ can affect primary osteoblast function on its own.

In addition to 1α-hydroxylase, osteoblasts express 24-hydroxylase [12], [13] and have the capability to synthesize 24R,25(OH)_2_D_3_ from 25(OH)D_3_ [14]. The metabolite 24R,25(OH)_2_D_3_ was originally thought to be inactive, however, several *in vivo* and *in vitro* studies support 24R,25(OH)_2_D_3_ bioactivity in bone tissue. In chickens, 24R,25(OH)_2_D_3_ in combination with 1,25(OH)_2_D_3_ treatment promotes fracture healing [15]. In addition, CYP24 knockout mice demonstrate a delayed fracture healing [16]. *In vitro*, 24R,25(OH)_2_D_3_ has positive actions on SV-HFO osteoblast differentiation by increasing ALP activity, osteocalcin secretion and matrix mineralization [17]. These findings suggest that primary human osteoblasts not only respond to the active metabolite 1,25(OH)_2_D_3_ but also to 24R,25(OH)_2_D_3_.

The aim of this research was to determine the effects of 25(OH)D_3_ on primary human osteoblast proliferation and differentiation, compared to 1,25(OH)_2_D_3_. To examine whether these effects of 25(OH)D_3_ occur through hydroxylation to 1,25(OH)_2_D_3_ we silenced CYP27B1 expression. However, osteoblasts synthesize not only 1,25(OH)_2_D_3_ from 25(OH)D_3_, but also 24R,25(OH)_2_D_3_ from 25(OH)D_3_. Therefore we hypothesized that the effects of 25(OH)D_3_ not only occurred through conversion to 1,25(OH)_2_D_3_ but also to 24R,25(OH)_2_D_3_.

## Materials and Methods

### Primary human osteoblast culture

Primary human osteoblasts were isolated from redundant trabecular bone fragments obtained from healthy donors undergoing pre-implant bony reconstruction of the mandible or maxilla with autologous bone from the anterior iliac crest. The donor group consisted of 11 males and 12 females with a mean age of 49.3±18.6 years. The protocol was approved by the Medical Ethical Review Board of the VU University Medical Center, Amsterdam, the Netherlands, and all donors gave their written informed consent.

A modification of the methods of Beresford and Marie [18], [19] was used. Shortly, the trabecular bone fragments were minced into small pieces and washed extensively with phosphate buffered saline (PBS). The bone pieces were treated with 2 mg/ml collagenase type II (300 U/mg; Worthington Biochemical Corporation, Lakewood, NJ, USA) for two hours in a shaking waterbath at 37°C. The pieces were placed in culture flasks with Dulbecco’s Modified Eagle Medium: Nutrient Mixture F-12 (DMEM/F12; GIBCO, Life technologies) supplemented with 10% Fetal Clone I (HyClone, Thermo Fisher Scientific), 100 U/ml penicillin and 100 µg/ml streptomycin (GIBCO, Life technologies), 1.25 µg/ml fungizone (GIBCO, Life technologies) and incubated at 37°C and 5% CO_2_. Medium was changed twice a week until cells reached confluence.

### Primary human osteoblast treatments

The vitamin D metabolites 25(OH)D_3_, 1,25(OH)_2_D_3_ and 24R,25(OH)_2_D_3_ were obtained from Sigma-Aldrich. Primary human osteoblasts were treated with or without different vitamin D concentrations as indicated in the figure legends.

To enable differentiation, primary human osteoblasts were cultured in osteogenic medium. Osteogenic medium consisted of complete medium with 10 mmol/L β-glycerophosphate (Sigma-Aldrich), 10 nmol/L dexamethasone (Sigma-Aldrich) and 50 µg/ml ascorbic acid (Sigma-Aldrich).

All experiments were performed in complete medium with 5% Fetal Clone I unless otherwise stated and all conditions, including treated and control groups, contained 0.1% ethanol.

### Proliferation

Primary human osteoblasts of the first passage were plated out in a 96 wells plate at a density of 4.000 cells/well. After 24 hours cells were exposed to medium with 25(OH)D_3_ (0, 100, 200 or 400 nmol/L) or 1,25(OH)_2_D_3_ (0, 1, 10 or 100 nmol/L). Medium was replaced every 3 days by complete medium with or without 25(OH)D_3_ or 1,25(OH)_2_D_3_. The proliferation of primary human osteoblasts was measured at day 3 and 6 using the XTT Cell Proliferation Kit (Roche Diagnostics) according to the manufacturer’s protocol. Briefly, cells were incubated with the XTT solution at 37°C, whereby the viable cells formed an orange formazan dye by cleaving the yellow tetrazolium salt XTT. After 2 hours the orange formazan solution was quantified by a photospectrometer (Berthold Technologies) at 450 nm.

### Differentiation

Primary human osteoblasts of the first or second passage were seeded into a 12 wells plate at a cell density of 40.000 cells/well. Cells were allowed to attach to the well for 24 hours before medium was changed to osteogenic medium with 25(OH)D_3_ (0 or 400 nmol/L) or 1,25(OH)_2_D_3_ (0 or 100 nmol/L). Medium was replaced every 3 or 4 days by complete medium with or without 25(OH)D_3_ or 1,25(OH)_2_D_3_. Culture medium was collected at day 3, 7, 10 and 14 of the differentiation culture and cell lysates were prepared for the measurement of osteoblast markers.

Procollagen type I aminoterminal propeptide (P1NP) was measured in culture medium using the UniQ PINP radioimmunoassay (Orion Diagnostica). The interassay variation was <8% over the whole concentration range.

ALP activity was measured in cell lysate that was made by scraping the cells in PBS-0.1% triton [12], and by sonificating of the lysate two times for 30 seconds at 50 Hz. ALP activity was measured by the ALP IFCC liquid assay (Roche Diagnostics), performed on a Modular analyzer (Roche Diagnostics). ALP activity was adjusted for total protein, measured by the BCA protein assay (Thermo Fisher Scientific) according to the manufacturer’s protocol.

Osteocalcin was measured in culture medium using an enzyme immunoassay (Biosource). Interassay variation was 15% at a level of 0.5 nmol/L, 8% at a level of 2 nmol/L and 9% at a level of 8 nmol/L.

### RNA isolation and real time RT-PCR

For RNA experiments primary human osteblasts of the first or second passage were seeded into a 12 wells plate at a cell density of 40.000 cells/well. Medium was changed after 24 hours and primary human osteoblasts were treated with different vitamin D metabolites as indicated in the figure legends. Total RNA isolation of primary osteoblasts was performed using the RNeasy Mini Kit (Qiagen) according to the manufacturer’s protocol. For removing residual DNA amounts an additional on-column DNA treatment was accomplished during the RNA isolation procedure. Total RNA concentration was measured with the Nanodrop spectrophotometer (Nanodrop Technologies).

RNA was reverse transcribed from 100 ng total RNA in a 20 µl reaction mixture containing 5 mmol/L MgCl_2_ (Eurogentec), 1X RT buffer (Promega), 1 mmol/L dATP, 1 mmol/L dCTP, 1 mmol/L dGTP, 1 mmol/L dTTP (Roche Diagnostics), 1 mmol/L Betaïne, 10 ng/µl random primer, 0.4 U/µl RNAsin (Promega) and 5 U/µl M-MLV RT-enzym (Promega). The PCR reaction of total 25 µl contained 3 µl cDNA, 300 nmol/L reverse and forward primer (table 1) and SYBR Green Supermix (Bio-Rad). The PCR was performed on an iCycler iQ Real-Time PCR Detection System (Bio-Rad): 3 minutes at 95°C, 40 cycli consisting of 15 seconds at 95°C and 1 minute at 60°C. The relative gene expression was calculated by the 2^−ΔCt^ method and TATA binding protein (TBP) was used as housekeeping gene.

**Table 1:** Primer sequence.

Gene	Primer sequence (5′- 3′)
CYP27B1	Forward: TGGCCCAGATCCTAACACATTT
	Reverse: GTCCGGGTCTTGGGTCTAACT
CYP24	Forward: CAAACCGTGGAAGGCCTATC
	Reverse: AGTCTTCCCCTTCCAGGATCA
Vitamin D receptor (VDR)	Forward: GGACGCCCACCATAAGACCTA
	Reverse: CTCCCTCCACCATCATTCACA
Alkaline phosphatase (ALP)	Forward: CCACGTCTTCACATTTGGTG
	Reverse: GCAGTGAAGGGCTTCTTGTC
Collagen type 1α1 (COL1α1)	Forward: GTGCTAAAGGTGCCAATGGT
	Reverse: ACCAGGTTCACCGCTGTTAC
Osteocalcin	Forward: GGCGCTACCTGTATCAATGG
	Reverse: TCAGCCAACTCGTCACAGTC
Osteopontin	Forward: TTCCAAGTAAGTCCAACGAAAG
	Reverse: GTGACCAGTTCATCAGATTCAT
TATA binding protein (TBP)	Forward: GGTCTGGGAAAATGGTGTGC
	Reverse: GCTGGAAAACCCAACTTCTG

### siRNA transfection

Silencing RNA was carried out to suppress CYP27B1 mRNA. Knockdown was performed using CYP27B1 SMART pool and the negative control ON-TARGET plus SMART pool (Thermo Fisher Scientific). Primary human osteoblasts of the first passage were electroporated with the Microporator Pipetype Electroporation System (Digital Bio, Hopkinton, MA, USA) using 1 pulse of 1200 V for 40 ms. After electroporation, 100.000 cells were seeded in a 24 wells plate in DMEM/F12 with 10% fetal clone I. Two days after electroporation of the cells, total RNA was isolated to determine CYP27B1 knockdown. Four days after the electroporation treatment, cells were incubated in complete medium with 25(OH)D_3_ (0 or 400 nmol/L) for 3 days. Complete medium was collected and stored at −20°C until 1,25(OH)_2_D_3_, 25(OH)D_3_ and 24R,25(OH)_2_D_3_ measurements. Cells were lysed and stored at −80°C until total RNA isolation.

### 1,25(OH)_2_D_3_, 25(OH)D_3_ and 24R,25(OH)_2_D_3_ measurements

Primary human osteoblasts were seeded into a 6 wells plate with a cell density of 500.000 cells/well. High bone cell density was used to raise the 1,25(OH)_2_D_3_ concentrations above the detection levels. After 24 hours, cells were incubated in medium consisting of DMEM/F12, 0.2% BSA, 100 U/ml penicillin, 100 µg/ml streptomycin, 1.25 µg/ml fungizone and 25(OH)D_3_ (0, 100, 200, 400 or 1.000 nmol/L). Medium was collected after 24 hours exposure of osteoblasts to 25(OH)D_3_.

The metabolite 1,25(OH)_2_D_3_ was measured in non-conditioned and conditioned medium using a radioimmunoassay (IDS). Cross reactivity with 25(OH)D_3_ and 24R,25(OH)_2_D_3_ was 0.1% and <0.01% respectively. Intra-assay variation was 8% at a level of 25 pmol/L and 9% at a level of 70 pmol/L, and interassay variation was 11% at a concentration of 25 and 70 pmol/L.

The metabolites 25(OH)D_3_ and 24R,25(OH)_2_D_3_ were analyzed in non-conditioned and conditioned medium using a liquid chromatography-tandem mass spectrometry (LC-MS/MS) method. Briefly, samples were incubated with deuterated internal vitamin D standards (d6–25(OH)D_3_ and d6-24R,25(OH)_2_D_3_) and protein-precipitated using acetonitrile. Supernatant was, after PTAD derivatization, purified using a Symbiosis online solid phase extraction (SPE) system (Spark Holland, Emmen, the Netherlands), followed by detection with a Quattro Premier XE tandem mass spectrometer (Waters Corp., Milford, MA). Intra-assay variation of 25(OH)D_3_ was 9.6%, 6.0% and 8.5% at a level of 58, 191 and 516 nmol/L, respectively. Intra-assay variation of 24R,25(OH)_2_D_3_ was 5.4% and 9.1% at a level of 46 and 150 nmol/L, respectively.

### Statistical analyses

Data were presented as mean ± SEM. Differences between 2 groups were assessed using Wilcoxon signed rank test. Differences between 3 or more groups were assessed using Friedman test followed by Dunn’s post hoc test. A p-value<0.05 was considered to be significant (*p<0.05, **p<0.01, ***p<0.001).

## Results

### Effects of 1,25(OH)_2_D_3_ and 25(OH)D_3_ on osteoblast proliferation

**Figure 1:**
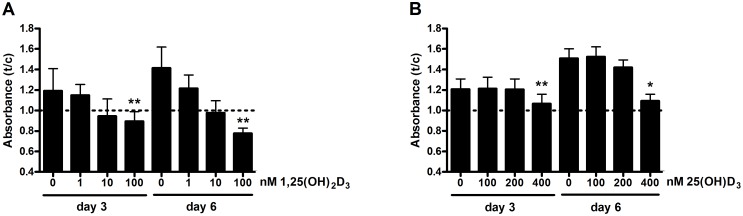
Effects of 1,25(OH)_2_D_3_ and 25(OH)D_3_ on primary human osteoblast proliferation. Osteoblasts were cultured in the presence of 0, 1, 10 or 100 nM 1,25(OH)_2_D_3_ (**A**) and 0, 100, 200 or 400 nM 25(OH)D_3_ (**B**) and the proliferation was quantified at day 3 and 6. Results (mean ± SEM) are expressed as treatment versus control ratios (time-point 0 was set at 1.0) using cells from 4 (A) or 7 (B) different donors. Results were analysed using Friedman test followed by Dunn’s post hoc test for each timepoint (*p<0.05, **p<0.01, ***p<0.001).

Primary human osteoblasts were cultured in the presence of 1,25(OH)_2_D_3_ or 25(OH)D_3_ for 6 days to compare the effects of these metabolites on the proliferation. Both 1,25(OH)_2_D_3_ (Fig. 1A) and 25(OH)D_3_ (Fig. 1B) significantly decreased the proliferation after 3 and 6 days of treatment. The reduction of the proportion of viable cells was found to be 28% (p<0.01) and 47% (p<0.01) in the presence of 100 nmol/L 1,25(OH)_2_D_3_ compared to control cultures at day 3 and 6 respectively. The metabolite 25(OH)D_3_ decreased the proliferation of primary human osteoblasts after 3 and 6 days of treatment at a concentration of 400 nmol/L. The reduction of the proportion of viable cells was 12% (p<0.01) and 28% (p<0.05) at day 3 and 6 respectively compared to control cultures.

### Effects of 1,25(OH)_2_D_3_ and 25(OH)D_3_ on osteoblast differentiation

**Figure 2:**
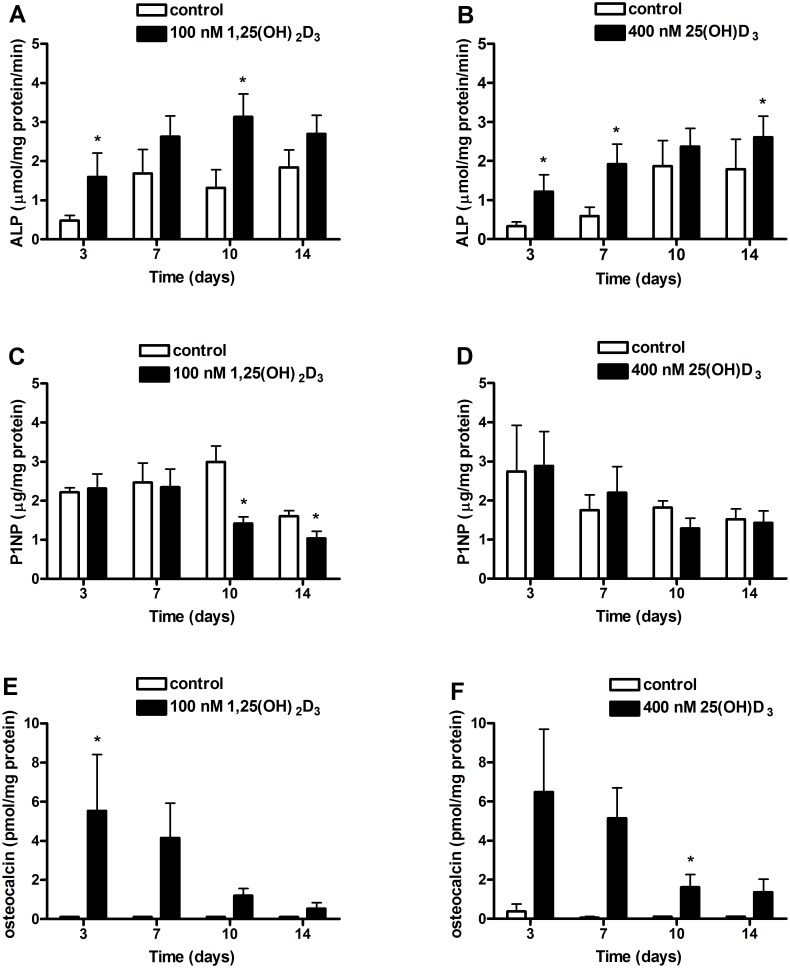
Effects of 1,25(OH)_2_D_3_ and 25(OH)D_3_ on ALP activity, P1NP and osteocalcin secretion by primary human osteoblast. Osteoblasts were cultured in the presence of 0 or 100 nM 1,25(OH)_2_D_3_ and 0 or 400 nM 25(OH)D_3_ and ALP activity (**A** and **B** respectively), P1NP (**C** and **D** respectively) and osteocalcin secretion (**E** and **F** respectively) were measured at day 3, 7, 10 and 14 of the differentiation. Results are expressed as mean ± SEM using cells from 5 different donors. Results were analyzed using Wilcoxon signed rank test for each timepoint (*p<0.05, **p<0.01, ***p<0.001).

Primary human osteoblasts were cultured in osteogenic medium containing 1,25(OH)_2_D_3_ or 25(OH)D_3_ for 14 days to compare the effects of these metabolites on the differentiation. Both 1,25(OH)_2_D_3_ (Fig. 2A) and 25(OH)D_3_ (Fig. 2B) stimulated the ALP activity during differentiation. The metabolite 1,25(OH)_2_D_3_ increased ALP activity at day 3 (332%; p<0.05) and 10 (238%; p<0.05) compared to control cultures. The metabolite 25(OH)D_3_ increased ALP activity at day 3 (369%; p<0.05), 7 (326%; p<0.05) and 14 (146%; p<0.05) compared to control cultures. P1NP secretion was decreased by 1,25(OH)_2_D_3_ (Fig. 2C) at day 10 (47%; p<0.05) and 14 (65%; p<0.05) compared to control cultures, but P1NP secretion was not significantly affected by 25(OH)D_3_ (Fig. 2D). Osteocalcin secretion was markedly enhanced by both 1,25(OH)_2_D_3_ (Fig. 2E) and 25(OH)D_3_ (Fig. 2F). The metabolite 1,25(OH)_2_D_3_ stimulated the secretion at day 3 (p<0.05), whereas 25(OH)D_3_ increased the secretion at day 10 (p<0.05).

**Figure 3:**
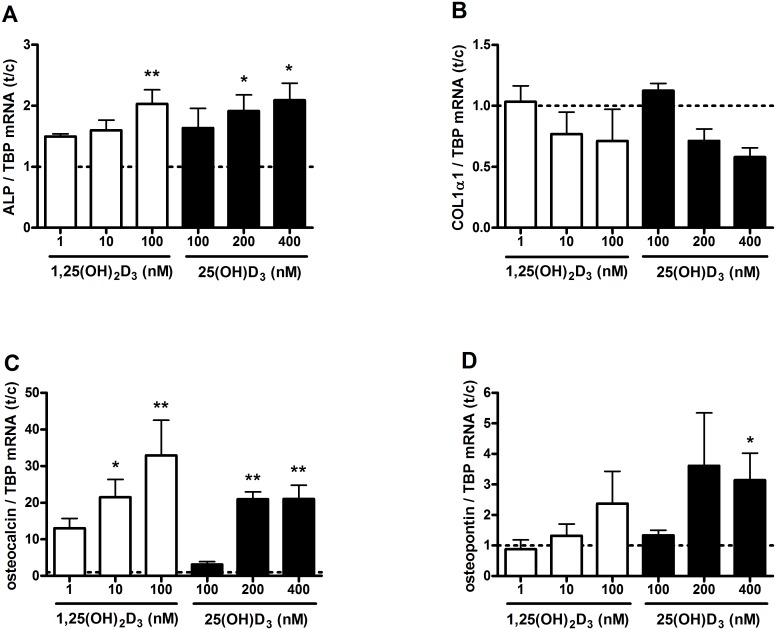
Effects of 1,25(OH)_2_D_3_ and 25(OH)D_3_ on mRNA levels of genes involved in primary human osteoblast differentiation. Osteoblasts were cultured in the presence of 0, 1, 10 and 100 nM 1,25(OH)_2_D_3_ or 0, 100, 200 or 400 nM 25(OH)D_3_ for 10 days in osteogenic medium and mRNA levels of ALP (**A**), COL1α1 (**B**), osteocalcin (**C**) and osteopontin (**D**) were determined. Results (mean ± SEM) are expressed as treatment versus control ratios (control was set at 1.0; dashed line) using cells from 5 different donors. Results were analysed using Friedman test followed by Dunn’s post hoc test (*p<0.05, **p<0.01, ***p<0.001).

Figure 3 demonstrates effects of 1,25(OH)_2_D_3_ or 25(OH)D_3_ on mRNA levels of genes involved in primary human osteoblast differentiation. ALP mRNA levels (Fig. 3A) were stimulated by both metabolites. The metabolite 1,25(OH)_2_D_3_ increased ALP mRNA levels at a concentration of 100 nmol/L (203%; p<0.01) and 25(OH)D_3_ increased ALP mRNA at a concentration of 200 nmol/L (191%; p<0.05) and 400 nmol/L (209%; p<0.05). Significant effects of 1,25(OH)_2_D_3_ or 25(OH)D_3_ on COL1α1 mRNA levels (Fig. 3B) were not observed. Osteocalcin mRNA was increased by 10 nmol/L (2147%; p<0.05) and 100 nmol/L 1,25(OH)_2_D_3_ (3289%; p<0.01) and by 200 nmol/L (2100%; p<0.01) and 400 nmol/L 25(OH)D_3_ (2102%; p<0.01). Osteopontin mRNA levels (Fig. 3D) were only significantly increased by 25(OH)D_3_ at a concentration of 400 nmol/L (314%; p<0.05).

### Effects of 1,25(OH)_2_D_3_ and 25(OH)D_3_ on VDR, CYP27B1 and CYP24 mRNA levels in primary human osteoblasts

**Figure 4:**
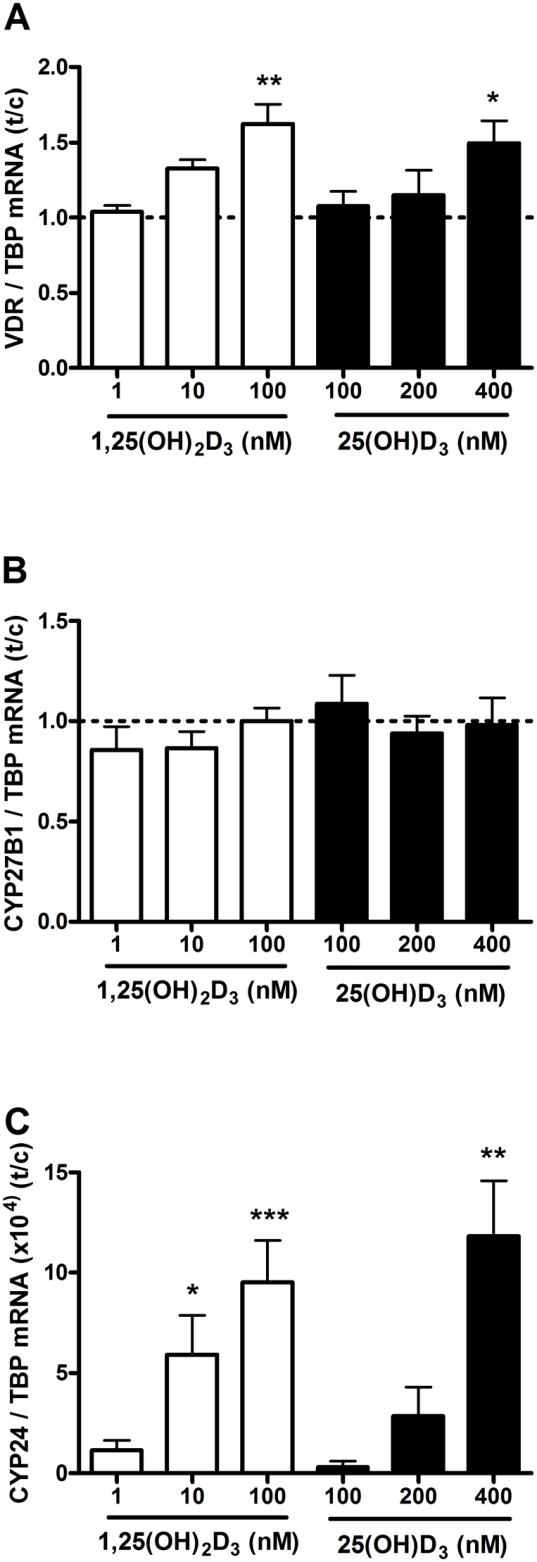
Effects of 1,25(OH)_2_D_3_ and 25(OH)D_3_ on VDR, CYP27B1 and CYP24 mRNA levels in primary human osteoblast. Osteoblasts were cultured in the presence of 0, 1, 10 and 100 nM 1,25(OH)_2_D_3_ or 0, 100, 200 and 400 nM 25(OH)D_3_ for 24 hours and mRNA levels of VDR (**A**), CYP27B1 (**B**) and CYP24 (**C**) were determined. Results (mean ± SEM) are expressed as treatment versus control ratios (control was set at 1.0; dashed line) using cells from 5 or 6 different donors. Results were analysed using Friedman test followed by Dunn’s post hoc test (*p<0.05, **p<0.01, ***p<0.001).

Proliferation and differentiation experiments showed that primary human osteoblasts were able to respond to 1,25(OH)_2_D_3_ and 25(OH)D_3_. Therefore we examined effects of both metabolites on mRNA levels of VDR, and metabolizing enzymes, CYP27B1 and CYP24. VDR mRNA levels (Fig. 4A) increased in the presence of 100 nmol/L 1,25(OH)_2_D_3_ (162%; p<0.01) and 400 nmol/L 25(OH)D_3_ (149%; p<0.05). CYP27B1 mRNA (Fig. 4B) did not respond to either 1,25(OH)_2_D_3_ or 25(OH)D_3_. CYP24 mRNA levels (Fig. 4C) increased dose-dependently in response to 1,25(OH)_2_D_3_ at a concentration of 10 nmol/L (p<0.05) and 100 nmol/L (p<0.001). In response to 25(OH)D_3_, a significant increase of CYP24 mRNA was found at a concentration of 400 nmol/L (p<0.01).

### Synthesis of 1,25(OH)_2_D_3_ and 24R,25(OH)_2_D_3_ by primary human osteoblasts

**Figure 5:**
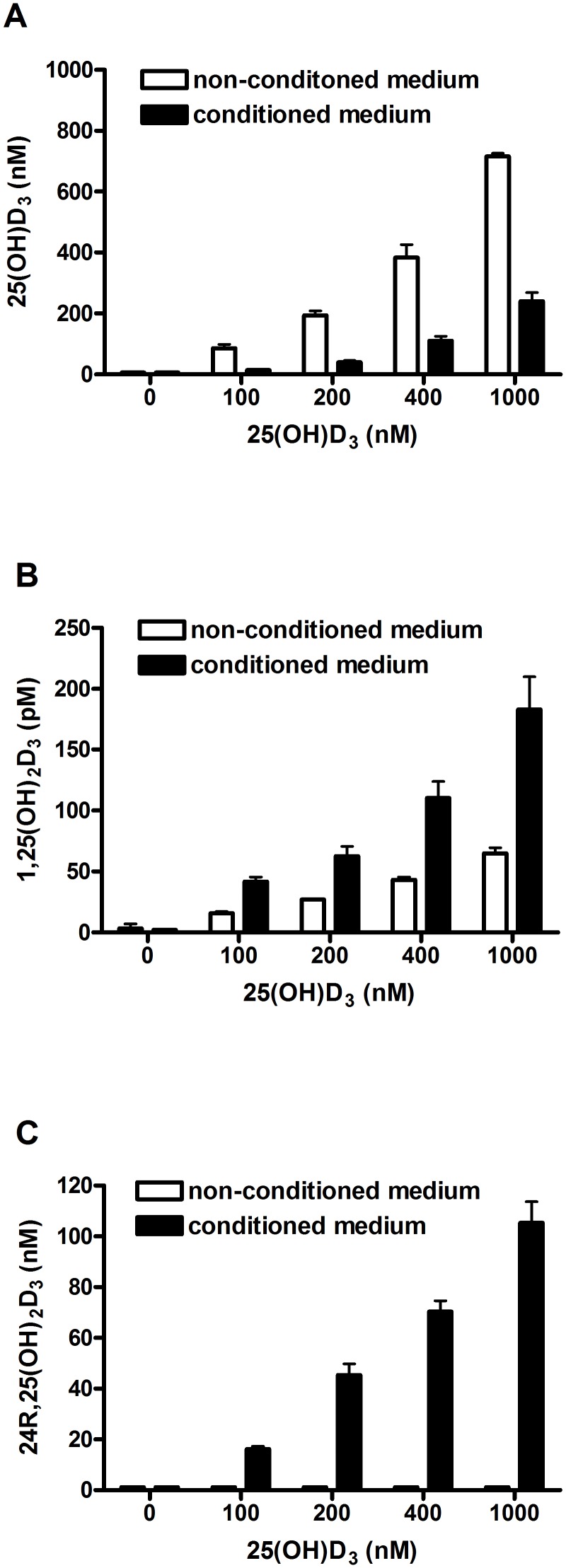
Synthesis of 1,25(OH)_2_D_3_ and 24R,25(OH)_2_D_3_ by primary human osteoblasts. Osteoblasts were cultured in the presence of 0, 100, 200, 400 and 1.000 nM 25(OH)D_3_ for 24 hours and 25(OH)D_3_ (**A**) 1,25(OH)_2_D_3_ (**B**) and 24R,25(OH)_2_D_3_ (**C**) levels were measured in non-conditioned and conditioned culture medium. Results are expressed as mean ± SEM using cells from 3 different donors.

Primary human osteoblasts were cultured in the presence of increasing concentrations of 25(OH)D_3_ to study the conversion to 1,25(OH)D_3_ and 24R,25(OH)_2_D_3_. After 24 hours incubation with 100, 200, 400 and 1.000 nmol/L 25(OH)D_3_, levels of 25(OH)D_3_ were strongly reduced to respectively 16%, 20%, 29% and 33% of non-conditioned values (Fig. 5A). The metabolite 1,25(OH)_2_D_3_ (Fig. 5B) was produced in a dose-dependent manner after 25(OH)D_3_ treatment. Mean concentrations of 1,25(OH)_2_D_3_ in medium were 8.8, 41.7, 62.3, 125.6 and 197.3 pmol/L after 24 hours incubation of cells with respectively 0, 100, 200, 400 and 1.000 nmol/L 25(OH)D_3_. In non-conditioned medium, 1,25(OH)_2_D_3_ concentrations ranging from 3.3–60.8 pmol/L were measured. The metabolite 24R,25(OH)_2_D_3_ (Fig. 5C) was also produced in a dose-dependent manner after 25(OH)D_3_ treatment. Mean concentrations of 24R,25(OH)_2_D_3_ in medium were <3, 16.1, 45.3, 70.2 and 105.4 nmol/L after 24 hours incubation of cells with respectively 0, 100, 200, 400 and 1.000 nmol/L 25(OH)D_3_. In non-conditioned medium, 24R,25(OH)_2_D_3_ was not detected (<3 nmol/L).

### Effects of 25(OH)D_3_ on mRNA levels of genes involved in primary human osteoblast differentiation after CYP27B1 silencing

**Figure 6:**
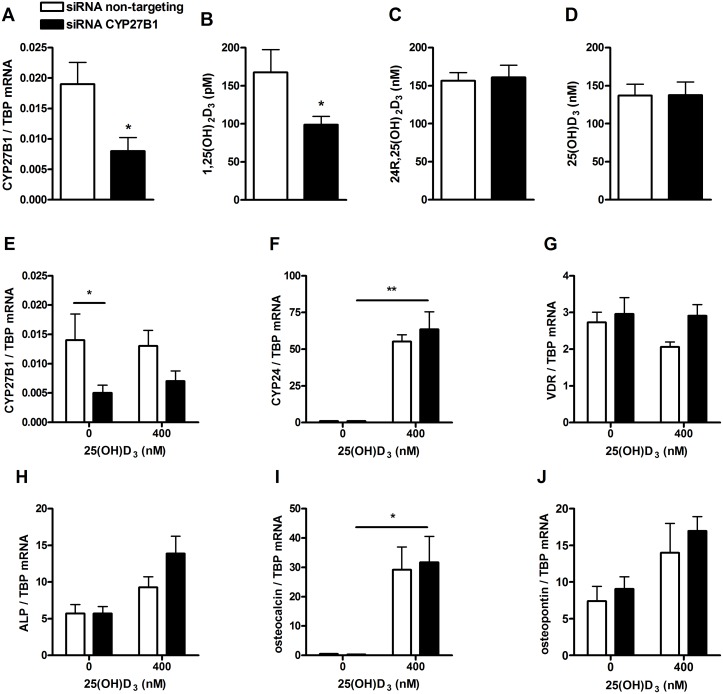
Effects of 25(OH)D_3_ on mRNA levels of genes involved in primary human osteoblast differentiation after CYP27B1 silencing. CYP27B1-silenced and control cells were incubated in the presence of 0 or 400 nM 25(OH)D_3_ for 3 days. CYP27B1 knock down was determined before 25(OH)D_3_ treatment (**A**). After 72 hours incubation with 25(OH)D_3_, we examined levels of 1,25(OH)_2_D_3_ (**B**), 24R,25(OH)_2_D_3_ (**C**) and 25(OH)D_3_ (**D**), and mRNA levels of CYP27B1 (**E**), CYP24 (**F**), VDR (**G**), ALP (**H**), osteocalcin (**I**) and osteopontin (**J**) in CYP27B1-silenced and control cells. Results are expressed as mean ± SEM using cells from 5 different donors. Results were analysed using Friedman test followed by Dunn’s post hoc test (*p<0.05, **p<0.01, ***p<0.001).

Silencing of CYP27B1 gene expression was used to examine whether 25(OH)D_3_ can directly act on osteoblast function. Treatment with CYP27B1 siRNA resulted in a 58% reduction of CYP27B1 mRNA compared to the control culture (p<0.05) (Fig. 6A). After 25(OH)D_3_ treatment, the reduction of CYP27B1 mRNA resulted in a decreased 1,25(OH)_2_D_3_ synthesis of 30% (p<0.05) compared to the control culture (Fig. 6B). Levels of 24R,25(OH)_2_D_3_ (Fig. 6C) and 25(OH)D_3_ (Fig. 6D) did not change in silenced and control cultures. After 72 hours of 25(OH)D_3_ treatment, the reduction of CYP27B1 mRNA was still 62% in the absence of 25(OH)D_3_ and 45% in the presence of 25(OH)D_3_ (Fig. 6E). No significant differences were seen between mRNA levels of CYP24 (Fig. 6F), VDR (Fig. 6G), ALP (Fig. 6 H), osteocalcin (Fig. 6I) and osteopontin (Fig. 6J) in control and CYP27B1-silenced cells that were exposed to 25(OH)D_3_.

### Effects of 24R,25(OH)_2_D_3_ on osteoblast differentiation

**Figure 7:**
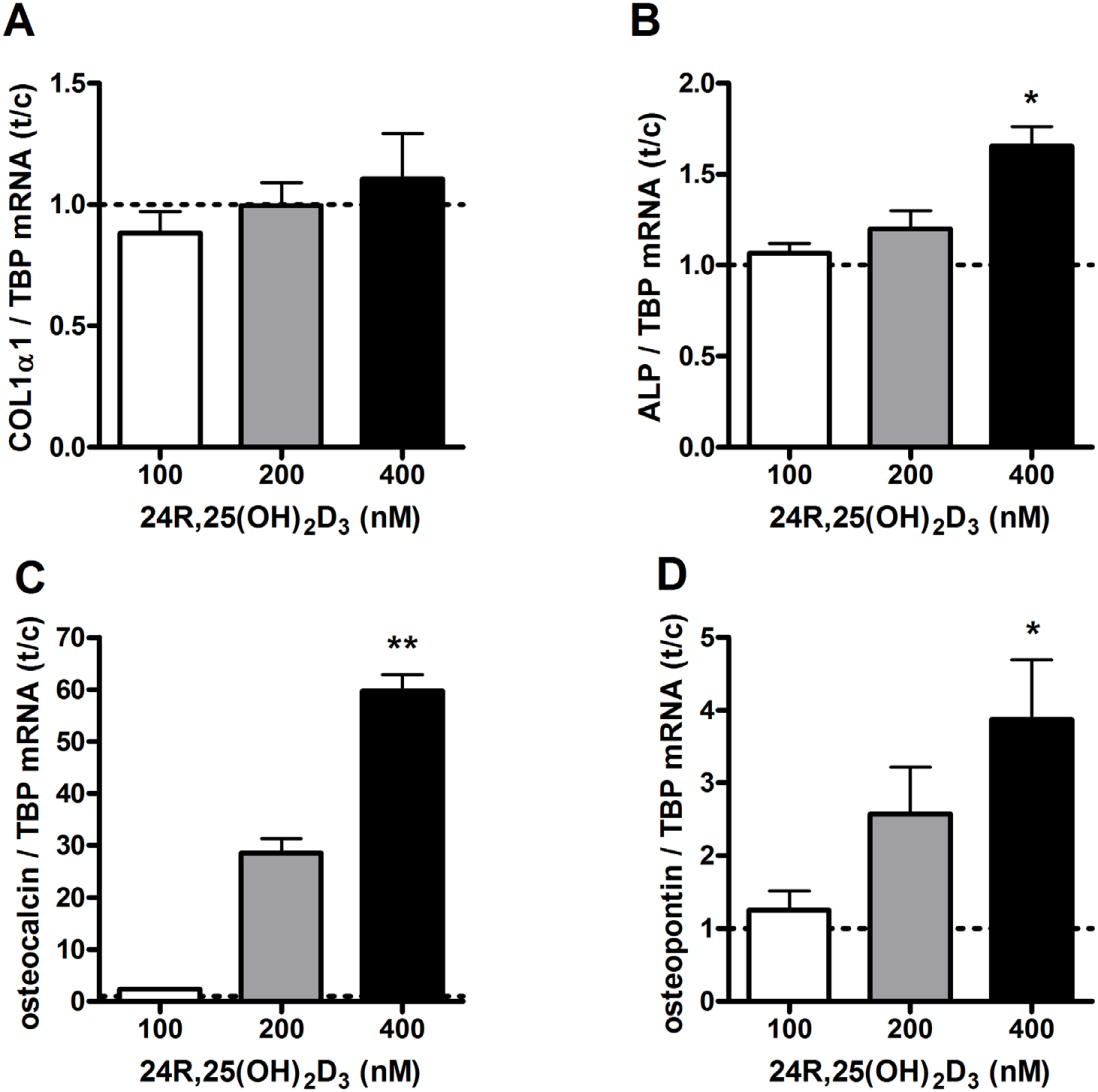
Effects of 24R,25(OH)_2_D_3_ on mRNA levels of genes involved in osteoblast differentiation. Osteoblasts were cultured in the presence of 0, 100, 200 or 400 nM 24R,25(OH)_2_D_3_ and mRNA levels of COL1α1 (**A**), ALP (**B**), osteocalcin (**C**) and osteopontin (**D**) were determined after 72 hours. Results (mean ± SEM) are expressed as treatment versus control ratios (control was set at 1.0; dashed line) using cells from 4 different donors. Results were analysed using Friedman test followed by Dunn’s post hoc test (*p<0.05, **p<0.01, ***p<0.001).

In addition to 1,25(OH)_2_D_3_, we showed that osteoblasts are able to synthesize 24R,25(OH)_2_D_3_ from the precursor 25(OH)D_3_. To examine whether 24R,25(OH)_2_D_3_ can act on osteoblast differentiation, primary human osteoblasts were cultured in the presence of 24R,25(OH)_2_D_3_. After 72 hours, 24R,25(OH)_2_D_3_ did not affect COL1α1 mRNA levels (Fig. 7A), but 400 nmol/L 24R,25(OH)_2_D_3_ increased ALP (137%; p<0.05) (Fig. 7B), osteocalcin (6182%; p<0.01) (Fig. 7C) and osteopontin (387%; p<0.05) (Fig. 7D) mRNA levels.

### Effects of 24R,25(OH)_2_D_3_ on VDR, CYP27B1 and CYP24 mRNA levels in primary human osteoblasts

**Figure 8:**
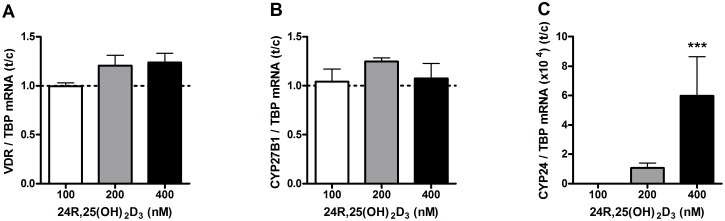
Effects of 24R,25(OH)_2_D_3_ on VDR, CYP27B1 and CYP24 mRNA levels in primary human osteoblasts. Osteoblasts were cultured in the presence of 0, 100, 200 or 400 nM 24R,25(OH)_2_D_3_ and mRNA levels of VDR (**A**), CYP27B1 (**B**) and CYP24 (**C**) were determined after 72 hours. Results (mean ± SEM) are expressed as treatment versus control ratios (control was set at 1.0; dashed line) using cells from 4 different donors. Results were analysed using Friedman test followed by Dunn’s post hoc test (*p<0.05, **p<0.01, ***p<0.001).

We did not observe effects of 24R,25(OH)_2_D_3_ on mRNA levels of VDR (Fig. 8A) and CYP27B1 (Fig. 8B). The metabolite 24R,25(OH)_2_D_3_ highly induced CYP24 mRNA (Fig. 8C) levels in cells treated with 400 nmol/L 24R,25(OH)_2_D_3_ (p<0.001).

## Discussion

This *in vitro* study shows the response of primary human osteoblasts to 25(OH)D_3_, 1,25(OH)_2_D_3_ and 24R,25(OH)_2_D_3_. Primary human osteoblasts responded to 25(OH)D_3_ by reducing their proliferation and enhancing their differentiation, similarly to 1,25(OH)_2_D_3_. We hypothesized that these 25(OH)D_3_ actions on osteoblast function occurred not only through hydroxylation to 1,25(OH)_2_D_3_, but possibly also through hydroxylation to 24R,25(OH)_2_D_3_. We could demonstrate that primary human osteoblasts expressed CYP27B1 and CYP24 and were capable to synthesize respectively 1,25(OH)_2_D_3_ as well as 24R,25(OH)_2_D_3_ from 25(OH)D_3_. Moreover, we showed that 24R,25(OH)_2_D_3_ increased mRNA levels of genes involved in primary human osteoblast differentiation.

The prohormone 25(OH)D_3_ has comparable effects to 1,25(OH)_2_D_3_ on growth and differentiation of primary osteoblasts. The metabolites 25(OH)D_3_ and 1,25(OH)_2_D_3_ reduced osteoblast proliferation and stimulated the differentiation as shown by increasing ALP activity (mRNA and protein) and osteocalcin secretion (mRNA and protein). Our study confirms previous studies in human osteoblastic cell lines and primary osteoblasts [12], [13], [20].

The effects of 25(OH)D_3_ on proliferation and differentiation likely occur through hydroxylation to 1,25(OH)_2_D_3_ [12], [13], since we and others demonstrated that osteoblasts are able to synthesize the active metabolite 1,25(OH)_2_D_3_ after exposure to the precursor 25(OH)D_3_ [12]–[14]. The consideration that effects of 25(OH)D_3_ occur through conversion to 1,25(OH)_2_D_3_ is supported by several *in vitro* blocking studies. In CYP27B1-silenced HOS cells, a human osteoblast cell line, it has been shown that exposure to 25(OH)D_3_ leads to a decline of osteonectin and CYP24 mRNA expression compared to control cells [21]. In human marrow stromal cells differentiated to osteoblasts, CYP27B1 is reported to be necessary for the antiproliferative and prodifferentiation effects of 25(OH)D_3_ [20]. Furthermore, in SV-HFO osteoblasts, ketoconazole almost complete blocked the effects of 25(OH)D_3_ on osteocalcin mRNA levels [12].

In our study, CYP27B1-silencing resulted in a decline of 1,25(OH)_2_D_3_ synthesis by primary osteoblasts. Despite this reduction, no significant differences in mRNA levels of differentiation markers were seen in CYP27B1-silenced cells compared to control cells after treatment with 25(OH)D_3_. It is likely that CYP27B1-silenced cells produced sufficient 1,25(OH)_2_D_3_ to induce a response. It is also possible that 25(OH)D_3_ affected osteoblast function through hydroxylation to 24R,25(OH)_2_D_3_. Levels of 24R,25(OH)_2_D_3_ were present in control and silenced cultures. Moreover, we showed that osteoblast cultures exposed to 24R,25(OH)_2_D_3_ had increased mRNA levels of ALP, osteocalcin and osteopontin. These results indicate a role for 24R,25(OH)_2_D_3_ in osteoblast differentiation. This is in line with previous research in the human osteoblast cell line SV-HFO in which 24R,25(OH)_2_D_3_ stimulated ALP activity and osteocalcin secretion by binding to the VDR [17]. Our results are also supported by a study in human mesenchymal stem cells, in which 24R,25(OH)_2_D_3_ enhances the osteoblastic differentiation by increasing ALP activity, osteocalcin mRNA levels and calcium mineralization of matrix [22]. In addition, our results are supported by a study in primary human osteoblasts that found increased osteocalcin production after 24R,25(OH)_2_D_3_ treatment [23]. However, due to incomplete CYP27B1 knockdown, 24R,25(OH)_2_D_3_ effects may be caused by 1α-hydroxylation to 1,24R,25(OH)_3_D_3_. The strong reduction of 25(OH)D_3_ levels in medium supports the idea that also other metabolites than 1,25(OH)_2_D_3_ and 24R,25(OH)_2_D_3_ are formed, for example 1,24R,25(OH)_3_D_3_. The metabolite 1,24R,25(OH)_3_D_3_ is able to enhance ALP activity, osteocalcin production and mineralization by SV-HFO osteoblasts [17]. In addition, 1,24R,25(OH)_3_D_3_ is even more potent than 24R,25(OH)_2_D_3_ [17].

In addition to the actions of 24R,25(OH)_2_D_3_ on mRNA levels of differentiation genes, 24R,25(OH)_2_D_3_ was also able to markedly enhance mRNA levels of CYP24. This may result in a higher production of 24R,25(OH)_2_D_3_ which suggests that 24R,25(OH)_2_D_3_ has the ability to regulate its own synthesis in a positive way (positive feedback). This is not in line with research performed in human mesenchymal stem cells differentiated to osteoblasts [22]. These osteoblasts decrease their CYP24 mRNA levels in response to 24R,25(OH)_2_D_3_ (at a concentration of 10 nmol/L) [22]. Added concentrations of 24R,25(OH)_2_D_3_ may explain the opposite results, since our results were obtained by using high 24R,25(OH)_2_D_3_ concentrations (400 nmol/L). Furthermore, we showed that 24R,25(OH)_2_D_3_, as well as 25(OH)D_3_ and 1,25(OH)_2_D_3_, had no effect on CYP27B1 mRNA levels. In human mesenchymal stem cells differentiated to osteoblasts, 24R,25(OH)_2_D_3_ (at a concentration of 10 nmol/L) decreases CYP27B1 mRNA and 1,25(OH)_2_D_3_ synthesis [22].

Actions of 24R,25(OH)_2_D_3_ can take place by activating the nuclear VDR [17], although the binding affinity of 24R,25(OH)_2_D_3_ to the VDR is 100 times less than 1,25(OH)_2_D_3_ [24]. In our study, 24R,25(OH)_2_D_3_ did not affect VDR mRNA, while 25(OH)D_3_ and 1,25(OH)_2_D_3_ increased VDR mRNA. Effects of 24R,25(OH)_2_D_3_ effects may be cell and concentration dependent, because at lower concentrations (10 nmol/L), 24R,25(OH)_2_D_3_ can decrease VDR mRNA and protein in human mesenchymal stem cells differentiated to osteoblasts [22].

The metabolite 25(OH)D_3_ itself may also be capable to activate the VDR and to subsequently affect mRNA levels of differentiation genes. This is supported by the *in vivo* study of Rowling et al [25] that supraphysiological levels of 25(OH)D_3_ can affect calcium and bone metabolism in the absence of its hydroxylation to 1,25(OH)_2_D_3_. In this study CYP27B1 knock out mice were fed a diet high in cholecalciferol which prevented hypocalcemia and almost rescued skeletal growth [25]. Several *in vitro* studies also support the hypothesis that 25(OH)D_3_ has direct effects on cells. Curtis [22] showed that 25(OH)D_3_ stimulates osteoblast mineralization in the presence of the cytochrome P450 inhibitor ketoconazole. Lou et al [26] showed that 25(OH)D_3_ is an agonistic VDR ligand and has direct inhibitory effects on proliferation in human LNCaP prostate cancer cells. In bovine parathyroid cells, 25(OH)D_3_ suppressed PTH secretion while 1α-hydroxylase was inhibited by clotrimazole. Therefore further studies are needed to clarify whether 25(OH)D_3_ can directly affect primary human osteoblasts. Although 25(OH)D_3_ may activate the VDR, the binding affinity to the VDR is less for 25(OH)D_3_ compared to 1,25(OH)_2_D_3_. Bouillon [24] reported that the binding affinity for 25(OH)D_3_ to the VDR is 50 times less than 1,25(OH)_2_D_3_.

In bone tissue, 25(OH)D_3_ metabolism may be beneficial since it is thought that locally synthesized 1,25(OH)_2_D_3_ supports osteoblast differentiation and matrix mineralization [12], [13], [27]. Serum 25(OH)D_3_ levels serve as substrate for local 25(OH)D_3_ metabolism and an adequate vitamin D status may therefore be essential [28]. In addition, low 25(OH)D_3_ serum levels in the range of deficiency may be a limiting factor for the synthesis of 1,25(OH)_2_D_3_ [29] and 24R,25(OH)_2_D_3_, and may result in reduced osteoblast differentiation and thereby a reduction of bone strength.

A limitation of this study is that complete blocking of the 1,25(OH)_2_D_3_ synthesis was not achieved in the RNA-silencing experiments. Therefore the question whether 25(OH)D_3_ itself is able to affect osteoblast function, can not be answered. Additional research is needed to achieve completely blocking of 1,25(OH)_2_D_3_ synthesis, for example studies with osteoblasts isolated from bone from CYP27B1 knock out mice. Furthermore, a critical point in our primary osteoblast cell culture model is the use of relatively high concentrations of 25(OH)D_3_, 1,25(OH)_2_D_3_ and 24R,25(OH)_2_D_3_ compared to normal serum levels in humans. Our concentrations of vitamin D metabolites were based on other studies in literature [12], [13], but effects of physiological levels of vitamin D metabolites on bone formation may be different. Lastly, in non-conditioned medium relatively high 1,25(OH)_2_D_3_ levels were measured. These levels of 1,25(OH)_2_D_3_ levels in non-conditioned medium are probably caused by cross-reactivity with 25(OH)D_3_ because of the high doses of 25(OH)D_3_ used in this study. However, our study clearly showed increased 1,25(OH)_2_D_3_ concentrations in conditioned medium compared to non-conditioned medium, which demonstrates the synthesis of 1,25(OH)_2_D_3_ by osteoblasts.

In conclusion, the vitamin D metabolites 25(OH)D_3_, 1,25(OH)_2_D_3_ and 24R,25(OH)_2_D_3_ can affect osteoblast differentiation directly or indirectly. The metabolite 25(OH)D_3_ is converted to both 1,25(OH)_2_D_3_ and 24R,25(OH)_2_D_3_, as demonstrated by measurements in culture medium. We showed that primary human osteoblasts not only respond to 1,25(OH)_2_D_3_, but also to 24R,25(OH)_2_D_3_ by enhancing the differentiation. This suggests that 25(OH)D_3_ can affect osteoblast differentiation via conversion to the active metabolite 1,25(OH)_2_D_3_, but also via conversion to 24R,25(OH)_2_D_3_ (direct or indirect via 1,24R,25(OH)_3_D_3_). Whether 25(OH)D_3_ has direct actions on osteoblast differentiation needs further investigation.
